# Can mechanical circulatory support be an effective treatment for HFpEF patients?

**DOI:** 10.1007/s10741-021-10154-1

**Published:** 2021-08-09

**Authors:** Einar Gude, Arnt E. Fiane

**Affiliations:** 1 Dept of Cardiology, Oslo University Hospital, Oslo, Norway; 2 Dept of Cardiothoracic Surgery, Oslo University Hospital, Oslo, Norway; 3 Faculty of Medicine, University of Oslo, Oslo, Norway

**Keywords:** HFpEF, MCS, Heart failure, Cardiac hemodynamics

## Abstract

Heart failure with preserved ejection fraction (HFpEF) is increasing in prevalence and represents approximately 50% of all heart failure (HF) patients. Patients with this complex clinical scenario, characterized by high filling pressures, and reduced cardiac output (CO) associated with progressive multi-organ involvement, have so far not experienced any significant improvement in quality of life or survival with traditional HF treatment. Left ventricular assist devices (LVAD) have offered a new treatment alternative in terminal heart failure patients with reduced ejection fraction (HFrEF), providing a unique combination of significant pressure and volume unloading together with an increase in CO. The small left ventricular cavity in HFpEF patients challenges left-sided pressure unloading, and new anatomical entry points need to be explored for mechanical pressure and volume unloading. Optimized and pressure/volume-adjusted mechanical circulatory support (MCS) devices for HFrEF patients may conceivably be customized for HFpEF anatomy and hemodynamics. We have developed a long-term MCS device for HFpEF patients with atrial unloading in a pulsed algorithm, leading to a significant reduction of filling pressure, maintenance of pulse pressure, and increase in CO demonstrated in animal testing. In this article, we will discuss HFpEF pathology, hemodynamics, and the principles behind our novel MCS device that may improve symptoms and prognosis in HFpEF patients. Data from mock-loop hemolysis studies, acute, and chronic animal studies will be presented.

## Introduction

Increasing numbers of patients are diagnosed with heart failure (HF), a disease with considerable impact on morbidity and mortality. It is estimated that 26 million patients suffer from HF and that 1–3% of adults and 10% of the population above the age of 70 years have HF. In the USA, approximately 6 million patients live with HF [1, 2]. Prevalence is expected to increase by almost 50% in the next 10 years due to an aging population, but also due to increased comorbidities among younger adults. HF with preserved ejection fraction (HFpEF) has been a focus of concern and is now more frequent among admitted patients than HF with reduced ejection fraction (HFrEF) [3, 4]. In HFrEF, left ventricular assist devices (LVADs) have successfully improved symptoms and survival. In this article, we will discuss HFpEF pathology, hemodynamics, and the potential for a novel mechanical circulatory support (MCS) device with unloading from the left atrium (LA) into different outflow sites in the arterial system that may improve symptoms and prognosis in HFpEF patients.

## HFpEF, more than a cardiac disease

The lack of a unified definition and treatment options has made the diagnosis of HFpEF challenging. HFpEF was previously characterized as diastolic dysfunction, typically after non-cardiac causes of dyspnoea were excluded in patients with normal ejection fraction (EF). Today, HFpEF is recognized as a syndrome comprised of multiple cardio-metabolic entities with cellular and inflammatory mechanisms leading to diastolic dysfunction, including biventricular systolic impairment, loss of atrial compliance associated with atrial arrhythmia, pulmonary vascular disease, loss of adequate chronotropic response, and finally hemodynamic derangement [5, 6]. Consequently, patients experience pulmonary and venous congestion secondary to high filling pressures and reduced cardiac (CO) output due to low stroke volumes (SV). HFpEF is more than a complex cardiac disease. It includes arterial hypertension and atherosclerotic disease, microvascular inflammation, renal failure (related to hemodynamic impairment, diabetes, and/or medications), and hepatic dysfunction due to congestion or fatty liver. Metabolic abnormalities include visceral adiposity with increased neurohormones and inflammatory cytokines and musculoskeletal degradation (either primary due to HF or secondary to metabolic syndrome and aging) [7]. Females are overrepresented [8]. Some patients experience chest wall restrictions due to loss of mobility. Predominant phenotypes differ across regions in Asia, the USA, and Europe. Whether a single common mechanistic pathway or multiple simultaneous processes lie behind this complex multi-organ pathological scenario is the subject of much discussion, but remains unanswered [9]. In a paper by Escher et al., HFpEF was divided into four phenotypes: (I) genetically inherited hypertrophic cardiomyopathy, (II) infiltrative cardiomyopathy with restrictive physiology, (III) nonhypertrophic cardiomyopathy, (IV) normal ejection fraction with comorbidities (i.e., hypertension, coronary artery disease) and hypertrophy. Dependent of phenotype, deranged hemodynamics caused elevated LA pressure and reduced CO consistent with HFpEF [10]. In this paper, the hemodynamics and physiology, independent of clinical phenotype, will be discussed together with the potential for targeted treatment with MCS in HFpEF.

Mortality in HFpEF is similar to HFrEF patients [11]. The causes of mortality are often more heterogeneous but represent the complexity and consequences of HFpEF and HF with secondary and/or simultaneous affection in other organs [12]. A mortality of 11% in 2.5 years (NYHA II–III) was demonstrated in the CHARM-preserved trial [13]. Owan et al. demonstrated an equivalent of 2-year mortality of 40% in both HFpEF and HFrEF among patients admitted with HF symptoms [11]. Amyloidosis, a special subtype of HFpEF caused by deposits of misfolded transthyretin in the extracellular space, has a poor prognosis, with median survival defined by the Gillmore classification between 24 and 69 months depending on levels of brain natriuretic peptide (BNP) above or below 3000 ng/l and estimated glomerular filtration rate below or above 45 ml/m [14]. Treatment for cardiac amyloidosis that directly targets the etiology of the disease is available, but unfortunately exclusively for this specific disease among the many etiologies behind HFpEF.

## Is HFpEF out of the reach of conventional HF treatment?

Several trials have tried to target treatment in HFpEF patients and have assessed the etiology of HFpEF (i.e., fibrosis), the hemodynamic derangement (increased filling pressure), or the symptoms (dyspnea, reduced functional capacity) with limited or no success. The European Society of Cardiology (ESC) stated in their HF treatment guidelines from 2016 that no HF medical treatment has demonstrated a reduction in mortality in HFpEF patients [15, 16]. The primary manifestations of HFpEF are shortness of breath and limited CO increase during exercise, which represent the treatment paradox in HFpEF. Preload reduction by diuretics improves shortness of breath but possibly reduces CO and thereby increases fatigue [17]. This is demonstrated in the Aldo-HF study, which demonstrated a reduction in pro-BNP and E/e (filling pressure in the LV/LA by echocardiography), but without effect on functional capacity (VO_2_) [18]. Beta-blockers improve left ventricle (LV) filling time, and also reduce heart rate (HR), which is almost linearly associated with CO, such that reduction may be unfavorable in HFpEF patients due to low SV related to small LV cavity size. On the other hand, reduced HR may improve diastolic filling by increasing the duration of diastole. In practice, the balance between unfavorable reductions in CO related to reduced HR and improvements in LV filling varies among individuals.

Beta-blockers are fundamental in HFrEF treatment independent of their effect on HR and have a positive effect on all-cause mortality in patients with EF < 40% [19]. HF with EF 40–49%, which was previously included in HFpEF trials is now defined as heart failure with mid-range ejection fraction (HFmrEF) while HFpEF is by ESC guidelines defined as ejection fraction (EF) > 50% [15]. Four major HFpEF trials (CHARM preserved, I preserved, PET-HF, and TOPCAT) all ended neutral in the primary endpoint [13, 20–22]. The potential treatment effect of aldosterone in the TOPCAT study and angiotensin/neprilysin inhibitors in the PARAGON study is limited to patients in sub-groups with EF ranging from 40–55% [23]. These results probably reflect targeting the systolic dysfunction in the HFmrEF more than the true HFpEF pathology. In the PARAGON trial, females and those with lower EF benefitted from angiotensin/neprilysin inhibitor compared to valsartan alone, highlighting the importance of defining normal EF. Normal EF in men is defined as above 50%, but normal EF in women is probably more than 50–55% [24]. In a study by Wehner et al., mortality demonstrated a *U*-shaped relationship to LVEF with a nadir risk corresponding to LVEF of 60–65% [25]. This might explain why women had the benefit of medical treatment at a higher EF than men and that the treatment effect was preferentially seen in a female population with poorer true systolic function than men, despite equivalent quantified EF [24]. Although these trials may suggest optimism, the data is too scant to define angiotensin-converting enzyme, angiotensin receptor blockers, and angiotensin/neprilysin inhibitors therapy as the salvation for HFpEF patients. The anti-diabetic drug sodium-glucose cotransporter-2 inhibitors, also called gliflozins, are promising in HFpEF and represent a completely new treatment strategy by altering the sodium/glucose channels and inhibit glucose reabsorption in the glomerular filtration.

The optimal treatment for a HFpEF patient would both reduce LA pressure and increase SV, but no such treatment is currently available. Interatrial shunt devices have been tested in HFpEF patients with a reduction of LA pressure and positive short-term outcomes on symptoms. However, these devices do not increase SV [26, 27]. Since many HFpEF patients are older and have a poor quality of life (QoL), endpoints in future HFpEF treatment trials should focus more on well-being in addition to mortality [15]. In HFrEF, LVADs have revolutionized treatment in very sick patients, but LV unloading is not suitable for the HFpEF group due to the small LV cavity size.

## How to target HFpEF with mechanical circulatory support?

LVADs have offered a new treatment alternative in terminal HFrEF patients with the unique combination of significant pressure and volume unloading in combination with an increase in CO, a win–win solution. Optimized and pressure/volume-adjusted solutions for HFrEF patients may conceivably be customized for HFpEF anatomy and pressure/volume curves.

MCS in HFpEF patients would likely involve alternative and potentially more technically difficult anatomical entry/inflow sites than the LV, with less robust anatomic structures and lower pressures than in the LV. The higher gradient between inflow and outflow will require more energy to maintain forward flow through the MCS. The side effects of LVADs, including systemic thromboembolism, bleeding, infections, and lack of pulse pressure, would have to be addressed to optimize MCS support in an elderly multimorbid HFpEF population. The placement of a possible MCS device in the HFpEF population must ideally be simplified and surgery minimalized, customized for a fragile patient group. In short, the ideal MCS device for the HFpEF population would be a low-risk, long-term durable, and easily implantable device for a population where destination therapy (DT) is most likely.

## Unloading of the LA—consequences for HFpEF hemodynamics

Burkoff et al. have elegantly demonstrated the hemodynamic effects of unloading LA to the aorta in ex vivo mock-loop simulation tests [28], while the use of The Tandem Heart device (Cardiac Assist Inc., Pittsburg, PA, USA) centrifugal pump in short-term unloading of LA to femoral artery has been proven clinically effective in HF patients by lowering LA pressure with additional systemic circulatory support [29]. For longer term use with the unloading of LA to the right axillary artery, Meyns et al*.* showed proof of concept with the partial support LVAD CircuLite Synergy Micro-Pump Device, later acquired by HeartWare (Framingham, Mass) and thereafter by Medtronic (Minneapolis, MN, USA) [30]. A novel impeller device, placed in the mitral position and intended to decrease LA pressure and increase LV preload, has been tested in a mock loop as a treatment principle [31]. A non-pulsatile pump in the mitral position will unload the LA in diastole due to pressure gradients between the LA and the LV; therefore, rotor speed (rpm) must be tuned to avoid retrograde flow through the pump in the systole. Escher et al. elegantly demonstrated the effect of a pneumatic pulsatile pump (CoPulse) in a mock loop with a single cannula in the LV in different HFpEF phenotypes. By CoPulsing with the heartbeat, filling and emptying the pump increased SV in addition to unloading from LV into the pump in diastole. CoPulse demonstrated the pump’s potential to unload the LA and increase CO differently for the four HFpEF phenotypes and is one of many potential treatment mechanisms relevant for the HFpEF population [10].

Timms et al. investigated the difference in hemodynamics with either LA or LV inflow cannulation in a HFrEF LVAD mock loop. EF, stroke work, and pump flow rates were lower with LA compared to LV cannulation in all HF conditions.

Adequate ventricular ejection remained with atrial cannulation under low levels of mechanical support, however, with risk of thrombus formation in very low EF [32].

Several studies have assessed the hemodynamic perturbations in HFpEF patients at rest and exercise, and understanding invasive properties is essential to unload the LA in HFpEF. LA pressures assessed by pulmonary capillary wedge pressure (PCWP) during right heart catheterization have been measured in healthy and in HFpEF patients [33]. In healthy individuals, LA pressure/PCWP varies from normal pressure at rest (5–12 mmHg) up to 20 mmHg at exhaustion. In a diagnostic exercise algorithm by Berry et al., exercise-induced PCWP > 20 mmHg and a parallel increase in mean pulmonary artery pressure (mPAP) (and a more or less unchanged transpulmonary gradient (TRP)) define exercise-induced post-capillary pulmonary hypertension (PH) [34]. Consequently, pulmonary vascular resistance may be normal, depending on the CO response to exercise. Long-standing HFpEF may induce structural changes in the pulmonary vasculature with secondary increased TPG, a complicating factor both in the diagnostic algorithm and the clinical approach to HFpEF treatment. Systolic pressure in the LA depends on atrial and ventricular properties and the degree of mitral regurgitation. V-wave in LA/PCWP can be caused by the direct pressure of LV contraction on the mitral leaflets or by a mitral regurgitation that also induces a volume load to the LA. Reddy et al. demonstrated V-waves in the LA up to 30 mmHg in patients with HFpEF during rest. During supine exercise with simultaneous PCWP measurements, mean pressure was measured up to 30 mmHg with a systolic V-wave up to 50 mmHg at a workload of only 20 W [35]. Atrial unloading should therefore be possible and clinically meaningful from a hemodynamic point of view.

In 2010, both Meyns and Klotz reported improved hemodynamic conditions with reduced pulmonary pressures and PCWP together with increased CO during partial unloading with the CircuLite pump (Medtronic) from the LA to the subclavian artery[30, 36]. CircuLite is a small non-pulsatile partial circulatory supporting pump with a capacity up to 3 L/m; consequently, the majority of the unloading presumably occurs during diastole, when the pressure gradient is at its lowest. Neither the effects on flow through the mitral valve and LV filling nor the volume circulating through the CircuLite pump is reported. The aortic flow signals are not reported, and theoretically, an increase in arterial pressure in diastole and a decrease in pressures in systole could be observed. The increase in the cardiac index from 2.0 ± 0.4 to 2.7 ± 0.6 L/min demonstrated a limited CO increase with a corresponding decrease in PCWP from 28.5 ± 6.0 to 19.7 ± 6.9 mmHg.

Despite the successful decrease of left side filling pressure and increased CO reported with the CircuLite, there are several pitfalls which have to be addressed.

**Fig. 1:**
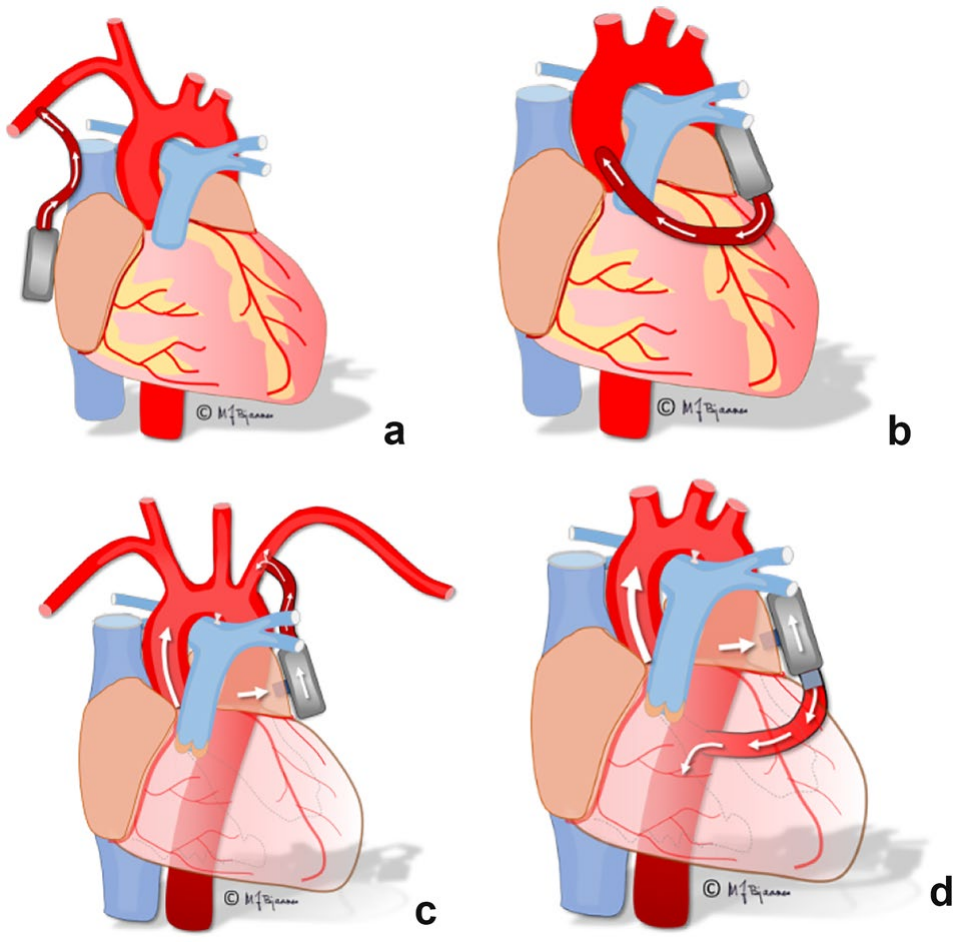
PulseVAD with pump inflow cannulation of left atrium posterior to groove between right and left atrium and outflow to right subclavian artery **a**, with pump inflow between left atrial appendage and left pulmonary veins and the outflow graft from the pump connected to ascending aorta **b**, left subclavian artery **c**, and descending aorta **d**. In all sheep studies described, we used strategy **d**

1. The LA must be partially unloaded to secure adequate LV filling, avoid LV thrombus, and secure adequate native antegrade aortic and especially coronary and carotid flow. The degree of LV filling and ejection and the need for forwarding aortic flow depends on the outflow graft position. LA unloading must therefore be less the more distant the outflow exit site is positioned. For example, outflow in the proximal ascending aorta as in today’s LVAD in HFrEF will secure adequate forward flow in ascending aorta including coronary arteries. Outflow position to the right subclavian artery as in the CircuLite secures antegrade carotid flow, but may cause stasis and turbulent flow in the area were antegrade aortic flow and exit graft flow meet. Outflow positioned in the left subclavian artery may compromise coronary perfusion if overpumping occurs and LV SV is too low. Outflow in the descending aorta requires controlled partial flow with possible fatal consequences in the case of low LV filling and aortic native flow and risk of hypoperfusion of the coronary and cerebral arteries. Favorably, outflow distant to the carotid arteries will reduce cerebral emboli from the pump, one of the major adverse events in traditional LVADs. Inflow and outflow sites determine the length of grafts, a possible modulator of graft thrombosis especially at low flow. Reduced embolus risk in the HeartMate III opens for aspirin-free anticoagulation in the ARIES HMIII trial (NCT 04,069,156). This approach would be even safer with an outflow graft distal to the carotid arteries. LVAD used as a right ventricular assist device with inflow in the right atrium (RA) and outflow to the pulmonary artery induces the possibility of minimal flow through the right ventricle (RV). Despite this dilemma, this approach has been successfully applied with less thrombosis using inflow in RA versus RV [37]. Contrary to systemic circulation, emboli to pulmonary circulation have far less severe consequences. Figure 1 demonstrates different outflow sites with inflow from the LA.
2. Unloading of the LA must overcome a higher gradient than traditional LVADs with inflow from the LV. Systolic pressure gradient from the LA (15–40 mmHg) to the aorta (100–140/50–100 mmHg) varies, depending on rest or exercise. Most importantly, however, the gradient to overcome is highest in systole, in contrast to traditional LVADs with inflow from LV where the gradient to overcome is highest in diastole. To overcome this gradient, a systolic ramp of pump speed in systole is required to overcome the gradient between inflow and outflow and to unload the greater part of the volume in systole as less unloading has to occur in the diastole. Pulsed MCS may reduce the risk of an embolus from the pump house, mimic natural circulation, and maintain the Windkessel effect which may be positive for end-organ and peripheral perfusion in DT patients [38].
3. LV filling occurs when the mitral valve is open in diastole. LA filling occurs in both systole and diastole, demonstrated by echocardiographic S and D flow in the pulmonary veins. LA unloading must preferably occur in systole when the mitral valve is closed. This will also minimize the hemodynamic negative effects of mitral regurgitation. Overpumping may interfere with mitral opening with possible adverse hemodynamic effects. An increase of pump speed in systole to overcome the inflow-outflow gradient and to unload the majority of volume must therefore be planned and timed in a pulsed algorithm.

## A new smartpump

**Fig. 2:**
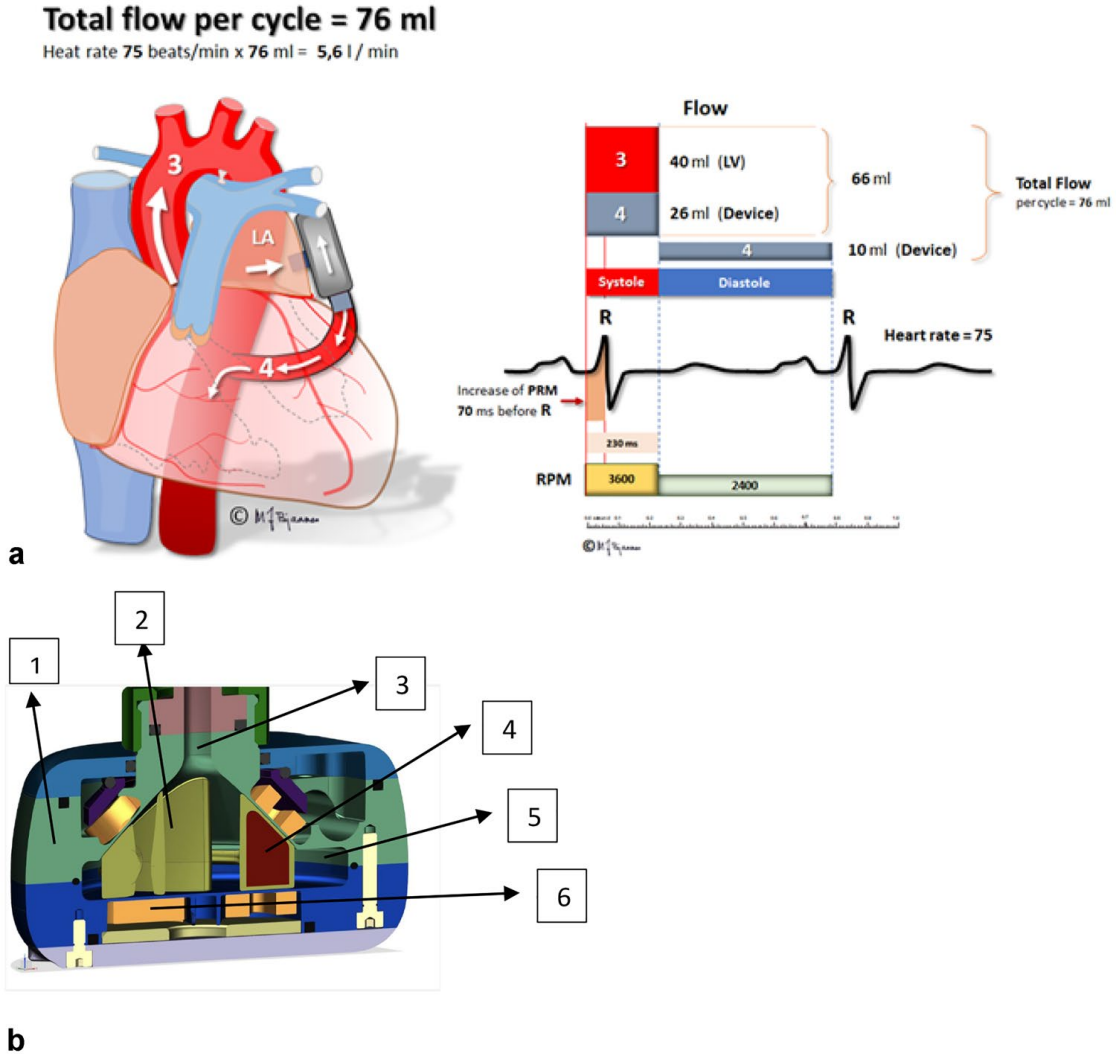
**a** Physiologic principle of unloading the left atrium to descending aorta. Increase in pump speed during systole to overcome the gradient from left atrium to aorta. **b** Cross-sectional graphic presentation of the PulseVAD. 1. Titanium casing. 2.  Hydrodynamically suspended rotor. 3. Inlet tract. 4.  Rare earth magnets.  5.  Outlet tract.  6. Motor coils

A recently developed pump, PulseVAD (NorthernResearch, Oslo, Norway), is a novel small pulsatile diagonal centrifugal rotary blood pump, using hydrodynamic suspension for magnetically elevated support of a four-blade impeller and partially unloading from the LA to the aorta (Fig. 2). The pump is adaptive to the physiological needs of the patients by use of epicardial ECG and sensor feedback algorithms. Increased pulsed power and increased pump speed in systole are programmed to unload the LA when the mitral valve is closed, maintain pulse pressure, and overcome the increased systolic gradient from the LA to the aorta (Fig. 2a). Cross-sectional animation of the pump is presented in Fig. 2b.

Pre-clinical implant testing was performed with human full blood (from six different healthy volunteer medical students) without anticoagulation in a closed ex vivo circuit with PulseVAD. Testing was performed six times during 8-h continuous runs with pulsed pump speed. Hemolysis parameters were analyzed with a simultaneous A and B test before starting the pump and repeated every hour including at test termination. Hemoglobin was targeted to 9–10 g/L. A gradient of 80 mmHg over the closed circuit was created with a tube clamp distal to the pump outlet. Pressure proximal to the pump was targeted to 10–15 mmHg, adjusted by a reservoir. During six tests, 76 time points were analyzed. Plasma-free hemoglobin (PfHb) was < 0.03 g/dL in 51/81 (63%) of the tests and remaining values were 0.04 ± 0.05 g/dL. Lactate dehydrogenase (LDH) was 110 ± 48 U/L, with no value above 250 U/L. Stable energy consumption less than 4–5 W was observed, with no development of heat. Pump flow was stable between 3 and 5 L/min. No malfunctions were observed.

Short- and long-term implantations in sheep were performed. Flow probes were attached to the proximal and distal aorta and the outflow graft of the pump. Intravenous lines demonstrated arterial and central venous pressures.

**Fig. 3:**
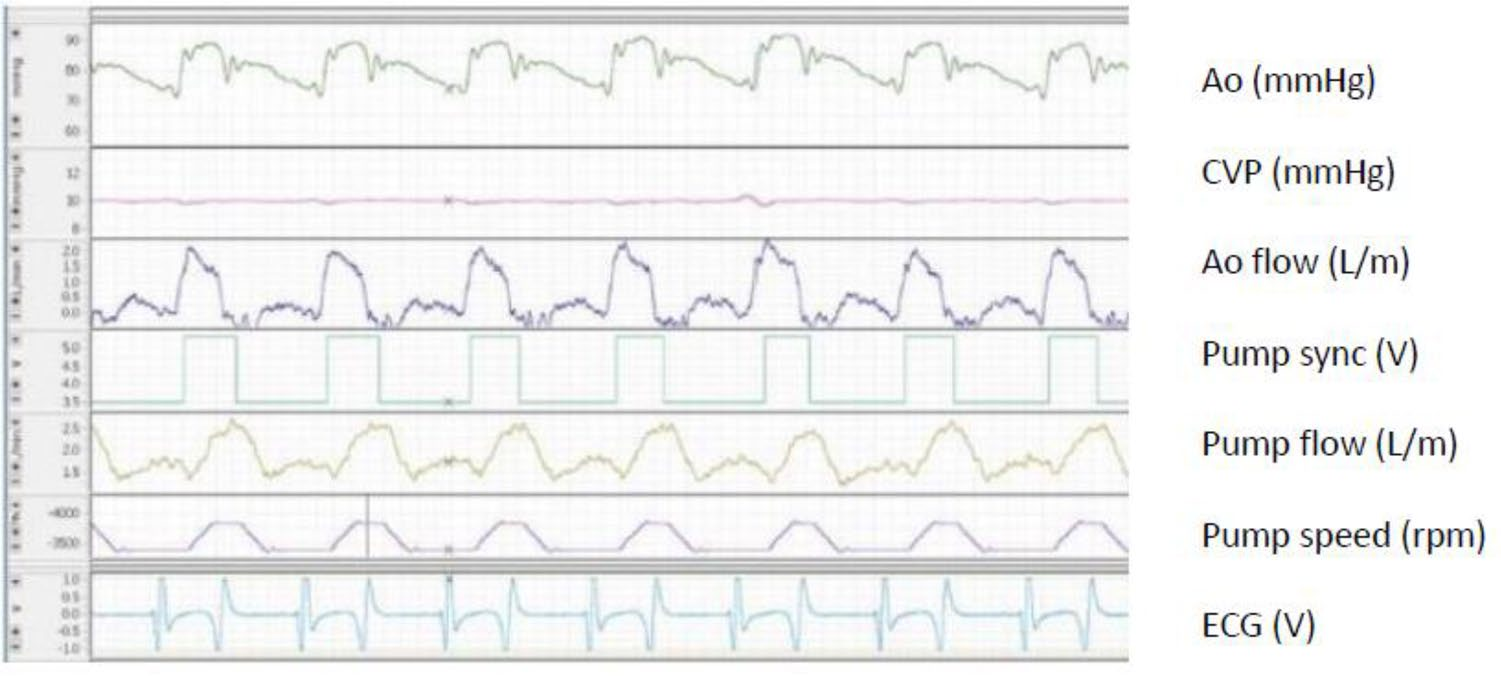
Hemodynamic and circulatory variables with PulseVAD in a sedated sheep, acute study. Pulsed and synchronized mode. Ao, aortic pressure (90/70 mmHg) measured invasively; CVP, central venous pressure (10 mmHg) measured invasively in internal jugular vein; Ao flow, aortic flow (0–2 L/m) measured with flow probe at proximal aorta proximal to pump outflow; Pump sync, pump synchronized to ECG; Pump flow (1.3–2.5 L/m) measured with flow probe at pump outflow graft; Pump speed (1800–3700 rpm); ECG, electrocardiogram (V)

Four short-term acute studies in sedated sheep were performed to test proof of concept. The physiology of the PulseVAD is demonstrated with aortic pressure and flow, pump speed, power, and flow from one of the studies (Fig. 3). Increased rpm during systole demonstrated increased systolic pump flow with a maintained aortic pulse pressure of 90/70 and flow measured with probes and iv lines proximal to the outflow graft. The timing of revolution per minute increase was adjusted to the R wave of the ECG to optimize the total aortic flow of approximately 5 L/min and systolic pressures approximately 100 mmHg. Both flow and pressure curves were affected by revolution per minute. Echocardiography was performed simultaneously and ideal optimal pump speed was when full aortic and mitral valve opening were observed. Variable revolution per minute was tested and no suction in the LA was demonstrated with systolic unloading and pump flow up to 4 L/min in stable sedated sheep. No supraventricular arrhythmias were observed during induced suction, and rapid loss of suction was demonstrated with a decrease of revolution per minute. Coronary and carotid artery flows were not measured during these tests.

In one long-term study (11 days), revolution per minute of 1900 and unloading of 2–2.5 L/m was demonstrated in the theater with sedated sheep. Blood pressure was 75/50 and pulse 90 sinus rhythms. No anticoagulation, anti-platelet drugs, or blood transfusion was administered during the 11-day follow-up. After waking up, the sheep were kept in a crib and monitored with a camera for 11 days without any observed adverse events. Pump parameters were kept unchanged for 11 days. No intravenous lines or flow probes were available during observations. Table 1 demonstrates stable pump speed (rpm), energy consumption (Watts), and biochemistry during the 11-day pump run. Autopsy (#9134, Leuven) demonstrated no signs of emboli or infarction in any organ, and pump inspection demonstrated no deposits in the pump house. Pump-atrium interface at implantation site connection was normal, and graft aorta interface was unremarkable.

**Table 1:** Pump parameters and biochemistry during 11 day pump study (absolute values ± standard deviation). *ALT*, alanine aminotransferase; *PfHb*, plasma-free haemoglobin

Day	Unity	Surgery	1	2	3	4	5	6	7	8	9	10
Haemoglobin	g/dL	11.5			12.0				11.4			12.5
Hematocrit		0.36			0.37				0.36			0.39
Creatinine	mg/dl	1.02			1.04				0.95			0.92
ALT	u/L	24.0			54.0				25.0			23.0
pfHb	mg/dl	9.00			< 5				< 5			< 5
Pump speed	rpm	1901 ± 120	1894 ± 114	1914 ± 190	1880 ± 101	1920 ± 101	1889 ± 131	1990 ± 130	1922 ± 117	1894 ± 99	1901 ± 139	1874 ± 144
Pump energy	Watt	3.5 ± 0.9	3.4 ± 0.8	3.6 ± 0.9	3.5 ± 0.8	3.6 ± 1.0	3.5 ± 0.9	3.6 ± 0.8	3.6 ± 0.7	3.5 ± 0.7	3.6 ± 0.8	3.6 ± 0.8

## Smartpump: surgical approaches for LA entry points and alternative arterial outflow sites

For inflow cannulation of the PulseVAD in HFpEF, the LA in humans may be cannulated via a minimally invasive surgical procedure by right anterolateral thoracotomy to access the groove between the RA and LA, by left anterolateral thoracotomy to access the lateral portion of the LA between left atrial appendage and left pulmonary veins, or by upper ministernotomy to access the roof of LA, between the superior vena cava and aorta. It is important to achieve stable positioning of inflow parts without risk of suction of the LA, as well as dislocation, kinking, or compression of the graft or nearby anatomical structures. The PulseVAD has a size and configuration which allows placement in a pocket beneath the pectoral muscle, outside the ribs on either side, or alternatively in the pleural cavity. The outflow graft from the PulseVAD may be connected to the right axillary artery, ascending aorta, left axillary artery, or descending aorta at different levels as demonstrated in Fig. 1. All of these surgical approaches have advantages and disadvantages with respect to surgical technique and risks, depending on the anatomy and frailty of patients.

An anterolateral thoracotomy left side was performed in all sheep studies with direct pump inflow cannulation of left atrium between the left atrial appendage and left pulmonary veins and with the outflow graft from the pump connected to descending aorta. In- and outflow from the smartpump in such a configuration may reduce the risk of cerebral embolization compared to other alternatives mentioned above.

## Conclusion

The HFpEF population is increasing in number and no therapy improving QoL and mortality is available. We have tested a new adaptive mechanical circulatory device with inflow from the LA and outflow to descending aorta. Initial animal tests have been promising, indicating that this approach may represent an effective long-term treatment for HFpEF patients with the potential to reduce filling pressures and increase cardiac output and thereby confer a positive effect on QoL.
